# Novel hepatocyte growth factor (HGF) binding domains on fibronectin and vitronectin coordinate a distinct and amplified Met-integrin induced signalling pathway in endothelial cells

**DOI:** 10.1186/1471-2121-6-8

**Published:** 2005-02-17

**Authors:** Salman Rahman, Yatin Patel, Jacqueline Murray, Kirti V Patel, Rushika Sumathipala, Michael Sobel, Errol S Wijelath

**Affiliations:** 1 Coagulation Research Laboratory, Division of Cardiovascular Medicine, GKT School of Medicine, St. Thomas' Hospital, London, UK; 2 Division of Vascular Surgery, University of Washington School of Medicine and VA Puget Sound Health Care System, Seattle WA, USA

## Abstract

**Background:**

The growth of new blood vessels in adult life requires the initiation of endothelial cell migration and proliferation from pre-existing vessels in addition to the recruitment and differentiation of circulating endothelial progenitor cells. Signals emanating from growth factors and the extracellular matrix are important in regulating these processes.

**Results:**

Here we report that fibronectin (FN) and vitronectin (VN) modulate the responses of endothelial cells to HGF (Scatter Factor), an important pro-angiogenic mediator. Novel binding sites for HGF were identified on both FN and VN that generate molecular complexes with enhanced biological activity and these were identified in the supernatants of degranulated platelet suspensions implicating their release and formation *in vivo*. In the absence of co-stimulation with an ECM glycoprotein, HGF could not promote endothelial cell migration but retained the capacity to induce a proliferative response utilising the Map kinase pathway. Through promoting Met-Integrin association, HGF-FN and HGF-VN complexes coordinated and enhanced endothelial cell migration through activation of the PI-3 kinase pathway involving a Ras-dependent mechanism whereas a Ras-independent and attenuated migratory response was promoted by co-stimulation of cells with HGF and a non-binding partner ECM glycoprotein such as collagen-1.

**Conclusions:**

These studies identify a novel mechanism and pathway of HGF signalling in endothelial cells involving cooperation between Met and integrins in a Ras dependent manner. These findings have implications for the regulation of neovascularization in both health and disease.

## Background

The generation and repair of blood vessels in adult life requires the regulation of endothelial cell survival, migration, proliferation and their differentiation from lineage-committed progenitors by the coordinated action of several classes of vaso-active agents including growth factors, cytokines, and the extracellular matrix (ECM) [1-4]. Elucidating the molecular mediators of these signals and their mechanism of action is vital to understanding the fine regulation of neo-vessel development and maintenance.

There is growing evidence pointing to a close collaboration between growth factors and the ECM in several biological processes including vasculogenesis and post-natal revascularization. Studies have shown that the response of cells to growth factors such as vascular endothelial growth factor (VEGF), platelet-derived growth factor (PDGF) and epidermal growth factor (EGF) are potentiated by integrin ligation to specific ECM glycoproteins [5-8]. In a previous report, we showed that VEGF-induced endothelial cell migration was augmented by fibronectin (FN) [9]. We also presented evidence that the VEGF/VEGFR-2 pathway is coupled to the integrin α_5_β_1 _through a mechanism involving the promotion of an integrin α_5_β_1_-VEGFR-2 signalling moiety generated as a consequence of receptor ligation by a VEGF-FN complex. These events promoted the sustained activity of Erk kinase, which was coupled to the migratory response. More recently, we presented data demonstrating that FN significantly enhanced VEGF-mediated migration of CD34^+ ^cells and their differentiation into endothelial cells [10]. In addition to the VEGF pathway, *in vitro *studies have highlighted the importance of hepatocyte growth factor (HGF) as a pro-angiogenic mediator. HGF, also termed scatter factor, has a well-established role in tumourogenesis but may be an important mediator of neovascularization since studies show that HGF induces the expression of VEGF in endothelial cells *in vitro *and that HGF synergises with VEGF to promote capillary-tube assembly in collagen matrices [11,12]. In addition, neovascularization in the rat cornea was also elevated by co-administration of HGF and VEGF compared to either growth factor in isolation [11]. The emerging significance of HGF as a pro-angiogenic mediator was further highlighted by a recent study of a large cohort of patients (1090 patients, CAPTURE trial) with acute coronary syndromes and identified serum levels of HGF as a positive indicator of patients' prognosis associated with a significantly lower event rate and increased collateralization of the target vessel [13]. Although the pro-angiogenic effects of HGF are known, the detailed mechanism of HGF action on the vascular cells, including the identity of intracellular mediators remains poorly understood.

In the present work we show that HGF forms a specific physical complex with FN and VN and that these complexes are present in degranulated platelet suspensions implicating a putative role *in vivo*. Significantly, we show that HGF-FN and HGF-VN molecular complexes induce a unique and enhanced intracellular signal employing Ras, thereby highlighting an important mechanism of growth factor receptor tyrosine kinase and integrin cooperation in promoting pro-angiogenic responses.

## Results

### Identification of novel HGF binding domains on FN and VN

**Figure 1:**
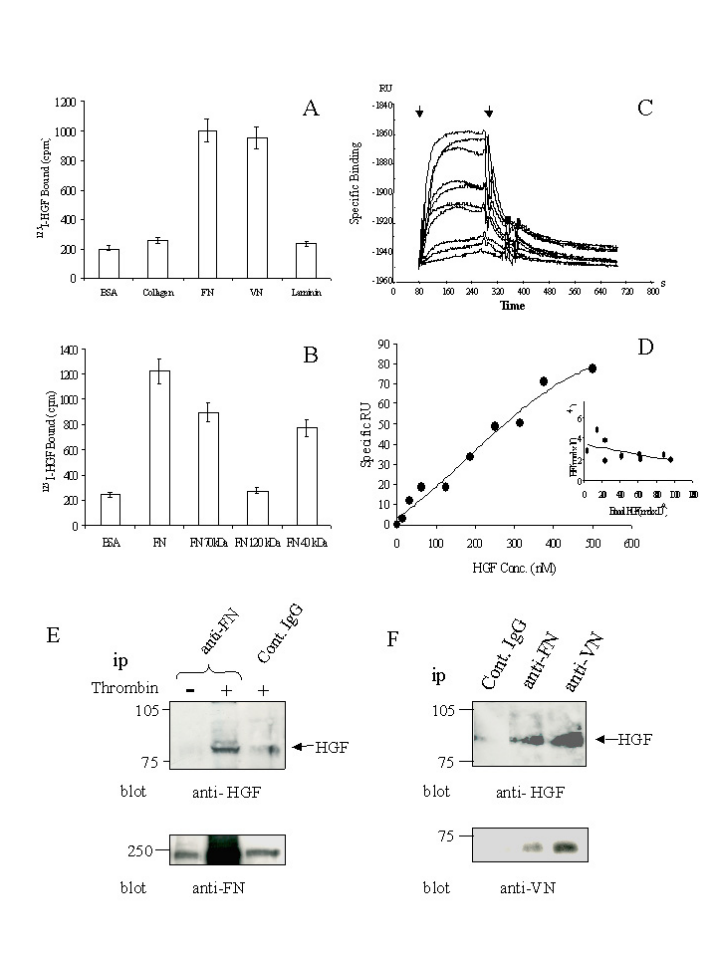
**Identification of HGF Binding Domains on FN and VN and the presence of HGF-FN and HGF-VN complexes in platelet supernatants. ***Panel A*-Plastic wells coated with various matrix proteins or BSA (10 μg/ml) were incubated with ^125^I-labelled HGF to equilibrium and then washed and counted in a γ-counter. *Panel B*-Plastic wells were coated with FN and FN derived proteolytic fragments or BSA. ^125^I-labelled HGF was incubated in these wells to equilibrium binding, washed and the bound HGF levels determined using γ-counter. *Panels C *&*D*-Binding of HGF to FN 70 kDa fragment in real time by SPR. *Panel C*-Real time binding isotherms of increasing concentrations of HGF (0, 16.5, 31.25, 62.5, 125, 188, 250, 315, 375, 500 nM)with association and dissociation phases for a single representative experiment with the arrows indicating injection start and finish. *Panel D*-The data from panel C is plotted as a function of HGF concentration (30–500 nM). Inset shows the data analysed by the method of Scatchard showing a single binding site with a K_dapp _= 300 nM. The data could also fit equally well to a two-site model (see text for details). n = 3. *Panel E*-Platelet suspensions (20.0 × 10^8^/ml) were stimulated with either saline (-) or 1 U/ml thrombin (+) for 5 min at room temperature to allow degranulation. Cellular and membranous material was cleared by centrifugation (100,000 × g) and the supernatants were immunoprecipitated with monoclonal antibodies to FN or an isotype matched control reagent (control IgG). Immune complexes were analysed by SDS-PAGE and Western blotting probing with an antibody to HGF (Santa Cruz). The blot was stripped and re-probed with antibodies to FN to confirm the specificity of the primary immunoprecipitation step. *Panel F*-Supernatants derived from thrombin-stimulated platelets were immunoprecipitated with antibodies with specificity for either FN, VN or an isotype matched control reagent. The immune complexes were analysed as in panel E. Lower panel shows the same blot stripped and re-probed with a monoclonal antibody to VN to confirm the primary precipitation. Blots were developed by chemiluminescence. The data shown are representative blots of experiments repeated three times.

We recently identified binding domains on FN for VEGF, which played an important role in promoting the activity of VEGF [9]. Since HGF is also an important angiogenic factor, experiments were designed to establish whether HGF had specific ECM binding partners. Using a solid phase assay, we measured the binding of ^125^I-labelled HGF to a variety of ECM molecules immobilized on plastic wells. As shown in Fig. 1A, ^125^I-labelled HGF bound to both FN and VN specifically with residual binding observed to either collagen-1 or laminin. Further experiments were performed to locate the HGF binding site on the FN molecule using purified FN proteolytic fragments immobilised onto the polystyrene microtiter wells. In these experiments ^125^I-labelled HGF bound to the 70 kDa N-terminal fragment and the 40 kDa C-terminal fragment. No significant binding was observed to the 120 kDa fragment that harbours the internal cell binding domain (Fig. 1B). To further analyse the association between HGF and FN, the interaction of HGF with the FN fragments was measured in real time by surface plasmon resonance analysis (SPR). As shown in Fig. 1C &1D, HGF bound to the 70 kDa N-terminal FN fragment immobilized on the sensor chip in a specific and saturable manner with a K_d _of approximately 300 ± 93 nM for a one-site model. The data shown in Fig. 1D could be applied to a two-site model with equal probability showing K_d _values for the high and low affinity sites of 15 nM ± 2 nM and 4 μM respectively. HGF binding to the 40 kDa fragment could not be measured directly by SPR, as immobilization of the 40 kDa fragment on the sensor chip appeared to mask the HGF binding site (data not shown).

### Platelets release HGF complexed to FN and VN

To establish whether HGF-FN and HGF-VN molecular complexes occur *in vivo *we examined platelets, a rich source of growth factors, for the presence of these complexes. Washed human platelet suspensions were stimulated with thrombin (1 U/ml) to promote degranulation and the derived supernatants were immunoprecipitated with antibodies directed to FN or VN. The resulting immune complexes were analysed for co-precipitation of HGF (Fig. 1E &1F). Immunoprecipitation of FN from thrombin-stimulated platelet supernatants resulted in significant co-precipitation of HGF (Fig. 1E). In contrast, minimal levels of HGF was observed in samples derived from unstimulated platelet supernatants or from samples derived from thrombin-stimulated platelet supernatants when an isotype-matched control antibody was employed in the experiment. Probing of the same blot with antibodies to FN confirmed that the primary precipitation of FN was responsible for the co-precipitation of HGF (Fig. 1E, lower panel). In a parallel experiment, immunoprecipitation of VN also co-precipitated HGF to a similar if not greater extent than FN (Fig. 1F). These experiments demonstrate that HGF is released from platelets and is found in the form of soluble molecular complexes with both FN and VN, confirming the results of the ligand binding studies *in vitro*.

### HGF-Induced endothelial cell migration is dependent upon co-stimulation with ECM

**Figure 2:**
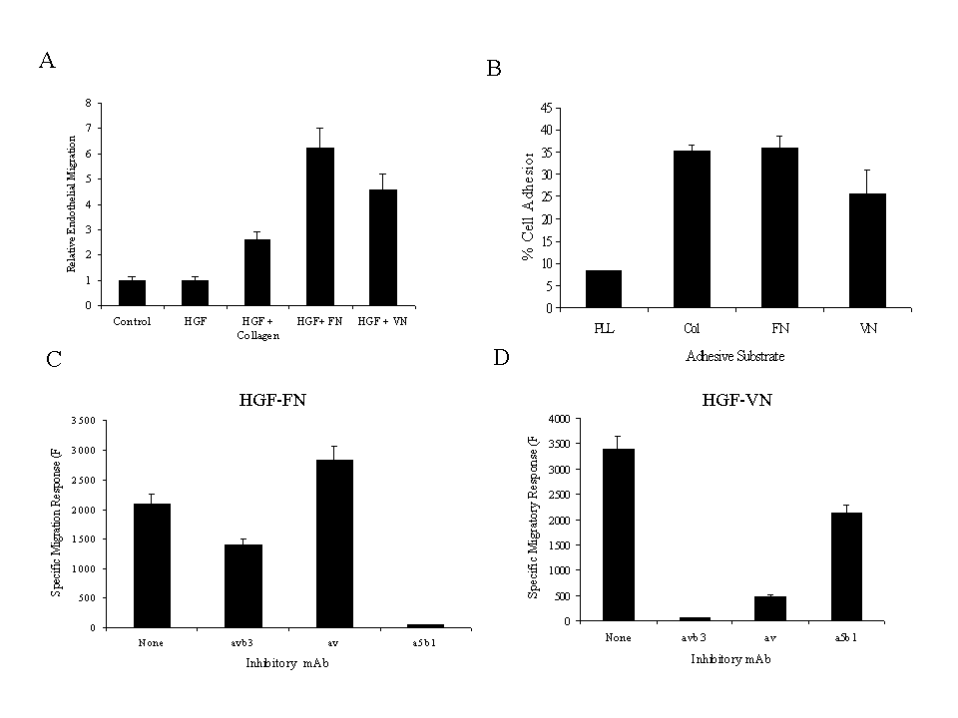
**Effect of ECM molecules on HGF induced Endothelial cell migration. ***Panel A*-Dye-loaded HMVEC suspensions (9 × 10^4^/ml) were treated with HGF (10 ng/ml) in the presence or absence of ECM molecules, FN or VN or collagen-1 in a modified Boyden chamber assay. Migration of cells was measured at 3 hours. The data is presented as relative migration with the migratory response presented as ratio to the basal migration in the absence of stimulus. *Panel B*-The levels of cell adhesion to Fluorblok transwell filters coated with either poly-L-lysine (PLL) or the various ECM glycoproteins was determined. Dye loaded HMVEC (10^5 ^cells) were treated with HGF (10 ng/ml) for 30 min prior to application to upper chamber of the transwell. Following extensive washes the number of adherent cells was determined using a fluorescence plate reader. The role and identity of integrins mediating the migration response in cells stimulated with HGF and FN (panel C) and HGF and VN (panel D) was demonstrated by the pre-treatment of HMVEC suspensions with anti-integrin monoclonal reagents (10 μg/ml) with specificity for integrins α_5_β_1 _(JBS5), α_v_β_3 _(LM609) and α_v_β_5 _(LM142) for 30 min at room temperature prior to application into the upper transwell chamber. The data is presented as specific migratory response in fluorescence units with the basal migration subtracted from the total migratory response. (n = 2).

We next sought to determine whether the responses of endothelial cells to HGF could be modulated by its ECM binding partners. In cell migration assays, human microvessel endothelial cells (HMVEC) were incubated with an optimal concentration of HGF alone or in combination with fixed concentrations of FN, VN or collagen-1 (Fig. 2A). Significantly, little or no endothelial cell migration above basal levels (control) was observed when cells were stimulated with HGF (10 ng/ml) in the absence of ECM. A moderate migratory response of endothelial cells to HGF was observed in the presence of collagen-1 (non-HGF binding ECM), which was less than 2-fold above basal levels. When HGF was co-administered with either FN or VN, endothelial cell migration was significantly enhanced by 4–5 fold. The differences in magnitude of the migration in the presence of these ECM glycoproteins was not related to variable degrees of cell adhesion upon the transwell filters as HGF-stimulated endothelial cells adhered equally well to ECM glycoprotein-coated transwells (Fig. 2B). The migratory response to HGF was dose responsive with a maximal response observed at a concentration of 10–20 ng/ml (data not shown). In addition, a negligible migratory response was observed when HMVEC were stimulated with these ECM molecules in the absence of HGF consistent with our previous report (data not shown, ref .9).

To further characterize the degree and identity of integrin involvement in the observed migratory response, we investigated the consequences of blocking integrin receptors on HMVEC with specific integrin antibodies prior to HGF-ECM stimulation. Antibodies directed to the integrin α_5_β_1 _completely inhibited HGF-FN-induced endothelial migration (Fig. 2C). In contrast, an antibody with specificity for the αv-subunit (LM142) had no inhibitory effect on endothelial cell migration. However, antibodies to the α_v_β_3 _integrin (LM609) did inhibit endothelial cell migration to HGF-FN by 20% suggesting an ancillary role for this integrin in mediating HGF-FN responses. When endothelial cell migration was induced by HGF-VN complexes, the integrin dependence shifted as expected (Fig. 2D). Under these conditions endothelial cell migration was predominantly dependent on αv-integrins for mediating the migratory signal with some apparent involvement of the integrin α_5_β_1 _(approximately 30%). This latter effect may be a consequence of integrin signal cross-talk (transdominant integrin regulation), as reported previously [14,15]. These experiments demonstrate that for HMVEC, HGF induced cell migration is dependent upon the ligation of integrins by ECM molecules.

### Met associates with α_v_β_3 _and α_5_β_1 _integrins

**Figure 3:**
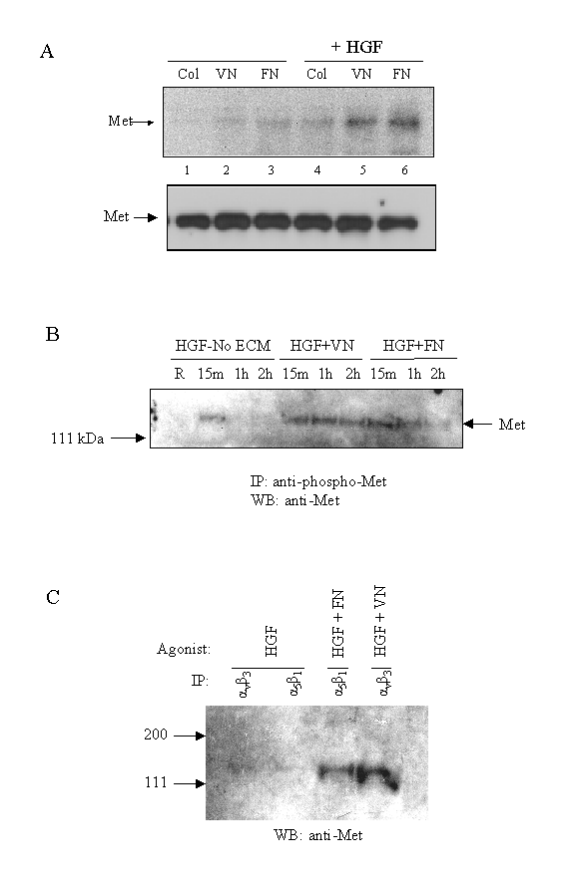
**Association of Met with integrins is driven by HGF-FN and HGF-VN complexes. ***Panel A*-HMVEC (1 × 10^6 ^/ml) were seeded onto collagen-1, FN or VN coated plastic wells and were stimulated with either saline or HGF (10 ng/ml) for 60 min at room termperature. Cells were lysed and the corresponding lysates (500 μg protein) were immunoprecipitated with antibodies to integrins α_2_β_1 _(lanes 1 & 4), α_v_β_3 _(lanes 2 & 5) and α_5_β_1 _(lanes 3 & 6). Immunocomplexes were analysed by SDS-PAGE and Western blotting probing with an anti-Met antibody using chemiluminescence detection (top panel). The bottom panel shows the antigen levels of Met in the corresponding cell lysates after analysis by SDS-PAGE (40 μg/lane) and Western blotting probing with an antibody to Met were essentially unaltered during the duration of the experiment. *Panel B*-HMVEC (5 × 10^6^) were stimulated with HGF or HGF-FN or HGF-VN complexes at the indicated time periods at room temperature. Cells were lysed and immunoprecipitated with a polyclonal antibody to phosphotyrosine Met and the immune complexes analysed by SDS-PAGE and Western blotting using a monoclonal antibody to Met. The Met antigen was detected using chemiluminescence. *Panel C*-HMVEC (5 × 10^6^) were stimulated with HGF or HGF-FN or HGF-VN complexes for 15 minutes at room temperature. Cells were lysed and the lysates immunoprecipitated with monoclonal antibodies to the integrins α_5_β_1 _and α_v_β_3 _and the immune complexes analysed by SDS-PAGE and Western blotting probing with a polyclonal antibody to Met.

Previous work [5-7,9] has demonstrated that the physical association of growth factor receptor tyrosine kinases and integrins promote enhanced cellular responses. We, therefore, postulated that the elevated cell migration induced by HGF-FN and HGF-VN in the present study could be due to a signalling mechanism involving the physical association between Met and integrins on endothelial cells. As shown in Fig. 3A, endothelial cell lysates derived from samples exposed to collagen-1, FN or VN, in the presence of HGF, when immunoprecipitated with antibodies to integrins α_2_β_1_, α_5_β_1 _and α_v_β_3 _respectively, predominantly co-precipitated Met with the integrins α_5_β_1 _and α_v_β_3_. In contrast, Met co-precipitation with the integrin α_2_β_1 _was minimal for lysates derived from cells stimulated with HGF and collagen-1. The level of Met expression in these samples was not altered by treatment of the cells with various combinations of HGF and ECM molecules (Fig. 3A lower panel) discounting the possibility that the differences in the level of Met co-precipitation was due to differences in the expression levels of its antigen. In the absence of HGF, co-precipitation of Met with the integrins α_5_β_1 _and α_v_β_3 _was minimal despite the presence of the ECM glycoprotein, indicating that ligation of the integrin with its cognate ligand was not sufficient to induce an association with Met.

To elucidate the role of Met activation in the formation of the Met-integrin signalling complex, endothelial cells were treated with HGF in the absence of ECM glycoprotein and with HGF-FN and HGF-VN complexes and the kinetics of Met tyrosine phosphorylation investigated (Fig. 3B). These experiments demonstrated that HGF in the absence of ECM glycoprotein could activate Met transiently with a strong signal present at 15 min but absent at 1 hour. In contrast, cells stimulated with HGF-FN and HGF-VN showed strong activation of Met at 15 min, which was sustained at 1 hour and was evident, although reduced, at 2 hours post-stimulation. Cell lysates derived from samples stimulated for 15 mins were also assessed for the presence of a Met-integrin complexes. As shown in Fig. 3C, HGF in the absence of FN or VN did not promote a significant association of Met with the integrins α5β1 or αvβ3. However, cells treated with HGF-VN and HGF-FN for 15 min contained significant levels of Met in a physical association with these integrins. These studies show that Met activation by HGF is insufficient to promote a physical association with integrins.

### HGF binding domains on FN and VN promote enhanced intracellular signals

**Figure 4:**
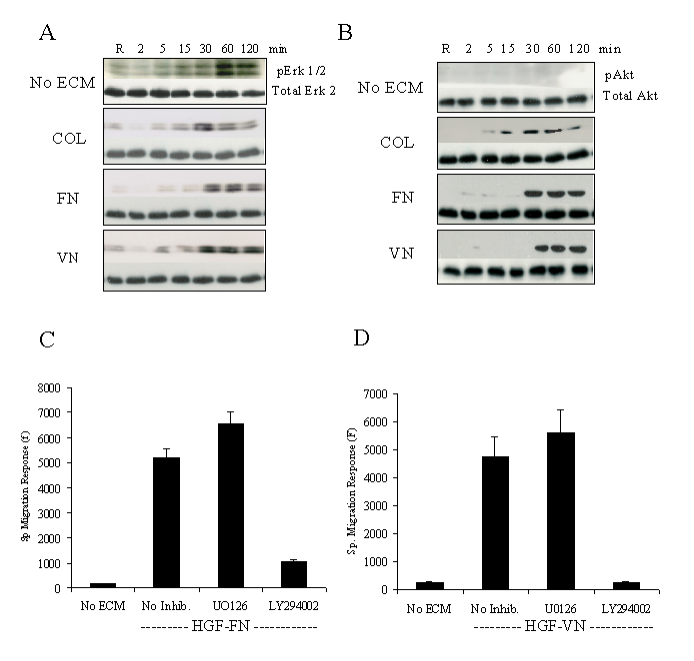
**HGF-FN and HGF-VN complexes augment HMVEC migration via the PI3 kinase pathway. ***Panel A*-HMVEC suspensions (1 × 10^6^/ml) were stimulated with HGF (10 ng/ml) in the absence of ECM molecules or in the presence of collagen-1, FN or VN (2 μg/ml) for varying time intervals (2–120 min) at room temperature. The reaction was stopped by rapid centrifugation and lysis of the cell pellet. Samples of cell lysate were analysed by SDS-PAGE and Western blotting probing with an antibody with specificity for phosphorylated Erk 1/2 (top panel). The blots were reprobed with an antibody to Erk 2 (bottom panel). *Panel B*-The samples from panel A were also probed simultaneously with an antibody with specificity for phosphorylated Akt (top panel) and these blots were stripped and re-probed with an antibody to Akt. Visualization was by chemiluminescence. *Panel C *&*D*-Effect of inhibitors on HMVEC migration in response to HGF-FN and HGF-VN respectively. The data is a representative experiment using triplicate samples which was performed three times giving essentially similar results.

We next investigated whether the association of Met with integrins modulated HGF/ECM-induced intracellular signalling, focussing on the ERK and the PI-3 kinase pathways. Analysis of the phosphorylation kinetics of Erk-1/2 in response to HGF alone or HGF/ECM combinations showed distinct patterns of activation (Fig. 4A). With HGF alone, Erk 1/2 phosphorylation showed kinetics with a peak signal at 60 min post-stimulation and significant reduction by 120 min although phosphorylation was still apparent. A distinct activation profile was observed when cells were stimulated with HGF and collagen-1 (non-HGF binding ECM), with Erk 1/2 levels peaking at 30 min and returning to near basal phosphorylation levels by 120 min. However, stimulation of endothelial cells with either HGF-FN or HGF-VN complexes promoted a rapid but sustained phosphorylation of Erk 1/2 with levels near maximal at 120 min post-stimulation. Analysis of the activation of the PI-3 kinase pathway was assessed by measurement of the phosphorylation status of Akt/PKB on Ser^473 ^(Fig. 4B). Interestingly, both distinct levels and kinetics of Akt phosphorylation were observed in these samples. When endothelial cells were stimulated with HGF in the absence of ECM co-stimulation, little phosphorylation of Akt above basal levels was observed. However, when cells were treated with HGF plus collagen-1, Akt phosphorylation was rapidly detected at 5 min and peaked at 30 min with significant reduction by 120 min. In contrast, with cells treated with either HGF-FN or HGF-VN complexes, Akt phosphorylation kinetics appeared to mirror Erk 1/2 phosphorylation kinetics implying a common regulatory mechanism for both pathways. As with Erk 1/2 phosphorylation, Akt phosphorylation peaked by 30 min post stimulation and this level of activation was sustained even at 120 min. Significantly, Akt phosphorylation levels in these samples were elevated approximately 3-fold, (assessed by densitometry) compared with the levels in observed in cells stimulated with HGF plus collagen-1 (data not shown).

### HGF/ECM-Induced endothelial migration is coupled to the PI-3 kinase pathway

To determine the intracellular pathway(s) that were coupled to the migratory response, HMVECs were treated with specific inhibitors of MEK and PI-kinase. In cell migration assays, LY294002 but not U1026 inhibited endothelial cell migration induced by HGF-FN (Fig. 4 C) and HGF-VN (Fig. 4 D), clearly demonstrating that the PI-3 kinase pathway was predominantly coupled to the migratory response and not the Map kinase pathway. Other inhibitors of potential down stream effectors were also tested. HGF-FN stimulated cells pre-treated with PP1, U73122, and piceatannol showed maximal migratory responses indicating that Src, PLCβ and Syk were not components of the migratory signal (data not shown).

### HGF-Induced endothelial proliferation is coupled to the Erk-pathway

**Figure 5:**
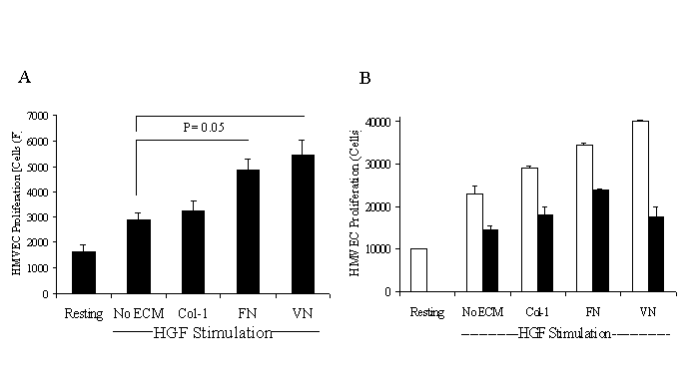
**HGF induced HMVEC proliferation requires the Map kinase pathway**. *Panel A*-HMVEC in MCDB-131 medium was plated on poly-D-lysine coated 48-well plates at a density of 2.5 × 10^3 ^cells/well and were stimulated with HGF (10 ng/ml) in the presence and absence of ECM molecules FN, VN or collagen-1. Basal proliferation was measured with cells treated with non-supplemented medium. Cell numbers were quantified 48 hours post-stimulation using CyQuant reagent. Data is presented as cell numbers with the increase of proliferation of HGF-FN and HGF-VN treated samples significant compared with cells treated with HGF alone as determined by a one-way ANOVA (p < 0.05) n = 3. *Panel B*-HMVEC were plated onto poly-D-lysine coated wells (1 × 10^4^/well) overnight in supplemented medium. Cells were then incubated with medium comprising 0.1% FBS plus HGF (20 ng/ml) in the presence or absence of ECM proteins (10 μg/ml) as shown containing no inhibitor (white bars) or the MEK inhibitor U1026 (10 μM, black bars). Cells numbers were quantified after a further 48 h using CyQuant reagent.

The effect of the co-administration of ECM molecules with HGF on endothelial cell proliferation was also investigated. In contrast to cell migration, HGF, in the absence of ECM molecules, induced a significant proliferative response (Fig. 5A). However, in the presence of FN or VN, HGF-induced endothelial proliferation was enhanced compared to HGF alone or in combination with collagen-1. As with the migratory response, endothelial cell proliferation was dose responsive to HGF with an observed maximal response at a concentration of 10–20 ng/ml (data not shown). Chemical inhibitors were then used to determine the signalling pathways involved in HGF-induced endothelial cell proliferation. In these studies, the MEK inhibitor, U1026 significantly impaired HGF-induced endothelial proliferation (50–80% inhibition) irrespective of co-stimulation with or without ECM molecules. This suggests that unlike migration, which was shown above, to be PI-3 kinase dependent, the Erk-pathway plays an important role in mediating HGF-induced endothelial cell proliferation. While both LY294002 and FPT-III blocked HMVEC proliferation, this appeared to be due to apoptosis (data not shown). This observation is consistent with the role of PI-3 kinase in promoting cell survival and a role for Ras in regulating PI-3 kinase in these cells [16,17].

### Ras is a specific, upstream regulator of Erk and PI-3 kinase pathways in cells stimulated with HGF-FN and HGF-VN complexes

**Figure 6:**
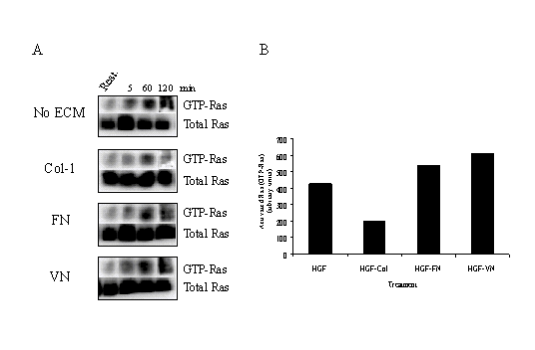
**Enhanced and sustained activation of Ras by HGF-FN and HGF-VN Complexes ***Panel A*-Kinetic GTP-Ras pull down analysis. HMVEC suspensions (3 × 10^6^/ml) were stimulated with HGF (10 ng/ml) in the absence and presence of ECM molecules (2 μg/ml) as shown for 5, 60 and 120 min at room temperature. Lane Rest. represents resting (unstimulated) levels of GTP-Ras. Cells were pelleted and lysed in cold MLB buffer (see methods). Samples of cell free lysates were incubated with of RBD-Sepharose and then analysed by SDS-PAGE and Western blotting probing for Ras. Visualization was by chemilumininescence using a Kodak imaging station. *Panel B*-The levels of GTP-Ras at the 60 min time point from the gel shown in panel A were quantified by densitometric analysis using ImageQuant software (Kodak).

**Figure 7:**
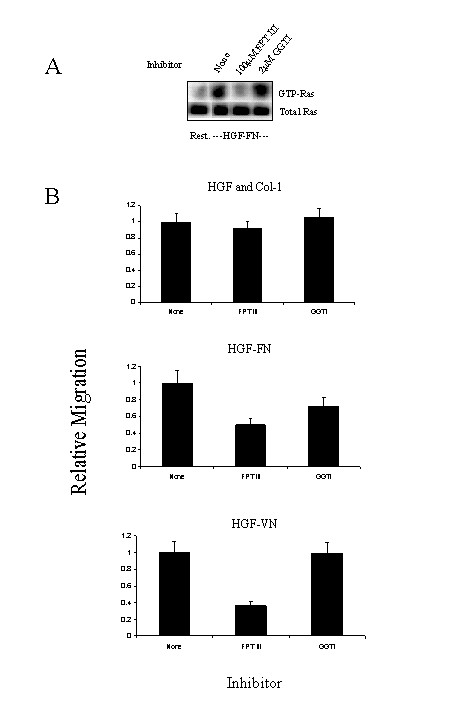
**HGF-FN and HGF-VN induced HMVEC migration is Ras dependent. ***Panel A*-GTP-Ras pull down assay showing the affects of FPT III (100 μM) and GGTI (2 μM) inhibitors upon GTP-Ras levels. HMVEC suspensions (3 × 10^6^/ml) were pre-treated with FPT III and GGTI inhibitors for 45 min at room temperature prior to stimulation with HGF-FN complexes for 60 min at room temperature. Samples were analysed as mention in the legend to Fig 2. *Panel B*-Effect of Ras inhibition upon cell migration. Calcein M loaded HMVEC suspensions (1 × 10^5^/ml) were pre-treated with the inhibitors as mentioned above prior to application to the top chamber of the transwell filter. Cells were stimulated with HGF plus ECM proteins (placed in the bottom chamber) as shown. Cell migration was measured at 3 hours post stimulation using a fluorescence plate reader. The data is shown as relative migration and is the combined data from two independent experiments with sample wells in triplicate.

The data shown above indicate that HGF-induced endothelial cell migration and proliferation were mediated by PI-3 kinase and Erk pathway respectively. We next investigated the role of Ras in regulating these two pathways induced by HGF-FN and HGF-VN complexes. Since Ras is a well-documented regulator of p85 PI-3 kinase and Erk and as well as a down stream effector of both the Met and integrin receptors, we assessed the activation status of Ras by measuring the comparative levels of GTP-loaded Ras after endothelial cells were stimulated with HGF in the presence and absence of ECM molecules (Fig. 6A &6B). Endothelial cells stimulated with HGF alone showed high levels of GTP-Ras at 60 min post-stimulation and this was sustained even at 120 min (Fig. 6A). In contrast, cells co-stimulated with HGF and collagen-1 showed activation of Ras at 60 min post-stimulation but to a significantly lower degree (approx 50% compared to HGF alone Fig. 6B), with the signal diminished by 120 min. With HGF-FN and HGF-VN co-stimulation, GTP-Ras levels were more than two-fold higher than observed when cells were co-stimulated with HGF-collagen-1 (Fig. 6B). Significantly, GTP-Ras levels were sustained at 120 min consistent with the observations of the activation profiles for the MAP kinase and PI-3 kinase pathways. These studies suggested that inhibiting Ras in cells stimulated with HGF-FN and HGF-VN complexes would exhibit reduced migration responses. To test this hypothesis, cells were treated with the membrane permeable farnesyltransferase inhibitor FPT-III, which inhibits Ras function as a consequence of the loss of membrane localization in the absence of farnesylation. Upon stimulation with HGF-FN, endothelial cells treated with FPT-III (100 μM) showed little activation of Ras following HGF-FN stimulation compared to basal levels in unstimulated cells (Fig. 7A). In comparison, pre-treatment of cells with the geranylgeranyl transferase inhibitor GGTI (2 μM) had little inhibitory effect on HGF-FN induced Ras activation. The effect of these inhibitors was tested in endothelial migration assays. Endothelial cells pre-treated with FPT-III displayed a profound reduction in cell migration of 50% and 73% when stimulated with HGF-FN and HGF-VN complexes respectively compared to cells pre-treated with GGTI (Fig. 7B). In contrast, FPT-III had little inhibitory effect on migration induced by HGF plus collagen-1 indicating that Ras has no significant role in the regulation of the migratory signal with this stimulus.

**Figure 8:**
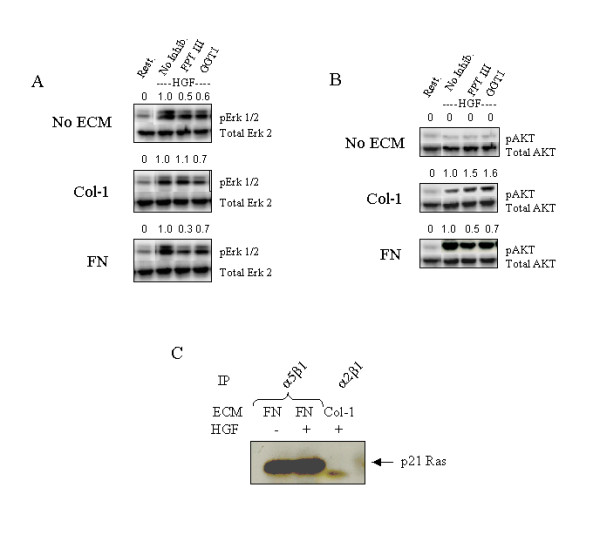
**HGF-FN Induced Activation of Map Kinase and PI-3 Kinase Pathways is Ras dependent ***Panel A *&*B*-HMVEC suspensions were pre-treated with buffer (no inhib.) or inhibitors FPT III (100 μM) and control GGTI (2 μM) for 45 min at room temperature prior to stimulation with HGF with no ECM proteins, or HGF plus collagen-1 or HGF-FN complexes for 60 min at room temperature. Cells were pelleted and lysed in an ice-cold lysis buffer. Samples were analysed by SDS-PAGE and Western blotting probing for phospho Erk 1/2 (panel A) and phospho AKT (panel B). The relative band intensities measured by image analysis software have been placed above each band as a ratio of the signal obtained in the sample where no inhibitor was present. Blots were stripped and re-probed with antibodies to Erk 2 and Akt to confirm equal loadings. *Panel *C-HMVEC suspensions (5 × 10^6^/ml) were incubated with either FN or collagen in the presence or absence of HGF as shown for 60 min at room temperature. Cells were pelleted and lysed in lysis buffer. Immunoprecipitation analyses of the lysates were performed with antibodies to integrins α5β1 and α2β1 and the immune complexes analysed by SDS-PAGE and Western blotting probing with antibodies to Ras. Visualization was by chemiluminescence.

To further characterise the role of Ras in regulating endothelial cell responses to HGF/ECM, the effect of the FPT-III inhibitor on the phosphorylation levels of Erk 1/2 and Akt was investigated. In cells stimulated with HGF alone, Erk 1/2 was activated and significantly inhibited by the FPT-III inhibitor and to a lesser extent by GGTI (Fig. 8A). A similar inhibitory profile was observed for cells stimulated with HGF-FN (Fig. 8A) and HGF-VN (not shown). In contrast, cells stimulated with HGF and collagen-1 showed no apparent reduction in Erk 1/2 phosphorylation levels when pre-treated with the FPT-III inhibitor suggesting little involvement of Ras in the activation of Erk 1/2. These samples were also assessed for Akt phosphorylation as an indication of PI-3 kinase activity. Consistent with our observations, stimulation of HMVEC with HGF in the absence of ECM did not lead to a significant activation of Akt (Fig. 8B). However, in the presence of collagen, Akt activation was observed but this was not affected by pre-treatment of the cells with FPT-III implying that Ras was not an upstream regulator of the activation of PI-3 kinase. In contrast, HGF-FN complexes promoted a 3-fold enhancement of Akt phosphorylation and this was inhibited by approximately 50 % by treating the cells with FPT-III (Fig. 8B). These observations suggest that the inhibition of Ras activation reduces the activation of PI-3 kinase for cells stimulated with HGF-FN complexes and not cells stimulated with HGF and collagen-1. The data would therefore predict that when HMVEC are stimulated with HGF-FN and HGF-VN complexes, specific integrins are utilized to recruit Ras, which in turn would regulate the activation of PI-3 kinase. To test this hypothesis, we immunoprecipitated integrins α5β1 and α2β1 from cells stimulated with HGF-FN and HGF with collagen-1 respectively and analysed these integrin immune complexes for the co-precipitation of Ras. High levels of Ras was specifically associated with α5β1 immune complexes and this appeared be independent of HGF stimulation. Little or no Ras was co-precipitated with the integrin α2β1 (Fig. 8C).

## Discussion

**Figure 9:**
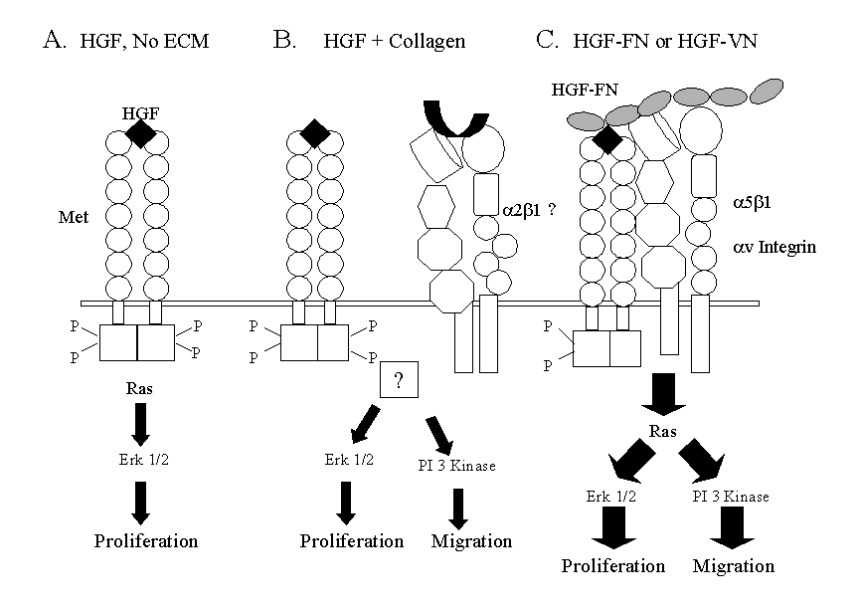
**Mechanisms of HGF induced cellular responses in endothelial cells. ***Panel A*-HGF in the absence of ECM molecules can activate the Map kinase pathway through the Met receptor tyrosine kinase leading to a proliferative response. *Panel B*-HGF and co-stimulation with collagen induces activation of both the Map kinase and PI-3 kinase pathways through an unknown mechanism presumably through integrin ligation, which is Ras independent. *Panel C*-HGF-FN (and by analogy HGF-VN) complexes promote enhanced cellular responses by promoting the association of integrins with the Met receptor leading to the recruitment and enhanced activation of Ras, Erk 1/2 kinases and PI-3 kinase promoting both elevated proliferative and migratory responses.

The major finding of the present report is that HGF-induced endothelial cell responses are significantly augmented through the formation of molecular complexes between this growth factor and the ECM glycoproteins FN and VN. The significance of this finding is highlighted by the observation that HGF-induced endothelial cell migration, a PI-3 kinase coupled response, did not occur in the absence of additional signals originating from the ECM. However, HGF-induced endothelial cell proliferation was evident in the absence of signals emanating from the ECM. These observations have led us to propose a model for the mechanisms of HGF-induced responses in endothelial cells (Fig. 9). This model shows that HGF alone can induce endothelial cell proliferation through its receptor Met via activation of the Ras-Erk kinase pathway (Fig. 9A). However, this signal is insufficient to promote significant cell migration for which an additional signal(s) from the ECM via specific integrin ligation appears necessary for activation of the PI-3 kinase pathway (Fig. 9B &9C). Uniquely, in cells stimulated with HGF-FN or HGF-VN complexes, which promotes the association of Met with integrins, an enhanced and unique intracellular signal is generated by the recruitment and sustained activation of Ras, which presumably, concomitantly activates both p85 PI-3 kinase and Raf (Fig. 9C). This is in contrast to the mechanism of activation of the PI-3 kinase pathway induced by HGF in the presence of collagen-1, which is Ras independent (Fig. 9B).

The model in Fig. 9C is supported by the following observations. The enhanced responses of HMVEC to HGF-FN and HGF-VN complexes is consistent with the observation that in these cells the activity of Ras, PI-3 kinase (AKT phosphorylation) and Erk 1/2 phosphorylation were sustained, and in the case of Ras and Akt, were 2–3 fold higher than observed in cells stimulated with HGF and collagen-1 (Fig. 6). The distinct signalling mechanisms induced by the co-activation of endothelial cells with HGF in the presence of a binding and non-binding ECM glycoprotein partner was also supported by the observation that treatment of cells with the inhibitor of Ras farnesylation, FPT III, reduced the phosphorylation of both Erk 1/2 and Akt kinases in cells stimulated with HGF-FN but not HGF plus collagen-1 (Fig. 8A &8B). Furthermore, Ras co-precipitated with the integrin α_5_β_1 _derived from endothelial cell lysates stimulated with HGF-FN complexes but not with the integrin α_2_β_1 _derived from cells stimulated with HGF and collagen-1 (Fig. 8C). These results are consistent with the pioneering work by Rodriquez-Viciana and colleagues who demonstrated the regulation of p85 PI-3 kinase by Ras via direct molecular interaction. It is now known that the regulatory subunit of all type 1, PI-3 kinases contain a Ras binding domain that associates with activated Ras (GTP-Ras) [17-20]. Therefore, our data and model demonstrating the sustained activation of Ras and PI-3 kinase by stimulation of endothelial cells with HGF-FN and HGF-VN complexes is consistent with previous work showing Ras to be a key regulator of PI-3 kinase. The identity of the Ras subtypes mediating the regulation of PI-3 kinase in our cell system is currently under investigation.

The results of the present study both support and extend our previous observations of the enhanced endothelial cell migration induced by VEGF-FN molecular complexes [9]. In that study, VEGF binding domains identified on FN drove the formation of VEGF-FN complexes that upon receptor ligation promoted the association of the integrin α_5_β_1 _with VEGFR-2. This co-receptor activation gave rise to a sustained activation of the Erk kinase activity, which promoted an enhanced migratory response. Similarly, the present work has shown that HGF-FN and HGF-VN molecular complexes induce the formation of Met-integrin signalling complexes promoting the transduction of a unique Ras-dependent signal. Several studies have illustrated the significance of the cooperation between integrins and growth factor receptor tyrosine kinases in mediating cellular responses. For example, the proliferation and migration of fibroblasts in response to PDGF-BB was enhanced in the presence of VN and was accompanied by the physical association of the α_v_β_3 _integrin with the PDGF-β receptor [5,7]. Furthermore, it was recently demonstrated that HGF in combination with FN prolongs the survival of GM-colony-forming cells [21] and enhanced the adhesion and motility of MTLn3 breast carcinoma cells [22]. In addition, integrins α_v_β_3 _and α_v_β_5 _were shown to be necessary for mediating FGF-2 and VEGF mediated angiogenesis respectively by the differential regulation of components of the Erk kinase pathway [23]. However, the present study extends these observations and is, to our knowledge, the first description of a distinct signalling pathway employed by the activity of growth factor-ECM molecular complexes as opposed to growth factors and ECM proteins functioning independently through ligation of their respective receptors. The identification of a Ras-dependent pathway in endothelial cells specifically activated with HGF-FN and HGF-VN complexes as opposed to HGF in the presence of collagen-1 is significant and correlates with Met-integrin association. Although the precise nature of the interaction between the Met tyrosine kinase and integrins was not elucidated, the role of Ras in this system appears important for the sustained and enhanced activation of the PI-3 kinase and Erk kinase pathways.

In contrast to the migratory signals promoted by VEGF-FN molecular complexes [9], HGF-FN and HGF-VN complexes induce a response in endothelial cells characterized by a tight coupling of the PI-3 kinase pathway to cell migration. Several additional pro-angiogenic mediators such as sphingosine 1-phosphate and NO, or the activation of CD40 and Eph B4 receptors by their counter ligands, promote endothelial cell migration through activation of the PI-3 kinase pathway [24-28]. In addition, HGF on its own was shown to stimulate smooth muscle cell migration in a PI-3 kinase dependent manner [29]. However, the lack of a significant migratory response, coupled with the absence of Akt phosphorylation observed in the present study, suggests that in primary endothelial cells the Met receptor is unable to activate PI-3 kinase without cooperative signals from the ECM/integrins. This observation is intriguing bearing in mind that Met has been shown to activate PI-3 kinase in epithelial cells via recruitment and activation of Gab-1, which directly interacts with the p85 subunit [30]. Consistent with our observation of an integrin dependency for signal transduction, Trusolino et al showed that in carcinoma cell lines Met induced signals were considerably amplified as a consequence of its constitutive association with the integrin α6β4. Intriguingly, in this system the authors showed that the role of the integrin α4 subunit was independent of extracellular integrin ligation since a truncated α4 construct lacking its extracellular portion could mediate HGF/Met responses and signals to downstream effectors provided that its ability to recruit the adaptor Shc was not affected [31]. In contrast, our studies using primary endothelial cells showed that integrin ligation was essential for generating a significant migratory signal via PI-3 kinase and in the case of HGF-FN and HGF-VN complexes, for promoting the association of Met with the integrins α_5_β_1 _and α_v_β_3 _respectively. Indeed, Met association with the integrins α5β1 and αvβ3 was dependent upon the activation of both Met and integrins through ligation of their cognate ligands since tyrosine phosphorylation of Met by HGF alone could not induce integrin association (Fig. 3C). These observations support the contention of a signalling mechanism requiring the formation HGF-ECM molecular complexes as a prerequisite for Met-integrin association and consequent signal amplifiation as proposed in Fig. 9C. However, the importance of integrin cytoplasmic domains in recruiting Ras and Ras-binding partners appears to reflect a common mechanism of HGF signal transduction between these cellular systems.

## Conclusions

The results of the present work demonstrate an important mechanism by which integrins collaborate with growth factor receptor tyrosine kinases on endothelial cells and predict that HGF binding domains on both FN and VN may play a significant role in promoting wound healing and post-natal neovascularization. In support of this contention, HGF-FN and HGF-VN complexes were identified in the supernatants derived from degranulated platelet suspensions indicating that these complexes do exist *in vivo *and may be deposited at sites of vessel perturbation or injury. This observation is similar to the identification of VEGF-FN molecular complexes in platelet supernatants in our previous report [9] and suggests that HGF and VEGF may act synergistically *in vivo*. Indeed, recent studies have shown that HGF synergises with VEGF to promote capillary-tube assembly in collagen matrices and neovascularization in the rat cornea [11]. Furthermore, HGF positively regulates VEGF expression and down regulates TSP-1, an inhibitor of angiogenesis, thereby promoting angiogenesis [32]. It is noteworthy that the HGF binding domains for FN were located in the same proteolytic fragments as those of VEGF, namely the N-terminal 70 kDa and C-terminal 40 kDa fragments. Further studies involving the fine mapping and characterization of the binding domains for VEGF and HGF on FN and VN should help decipher the mechanism of interplay between these important pro-angiogenic mediators.

## Methods

### Solid phase assay and Surface Plasmon Resonance Analysis (SPR)

ECM proteins and FN peptides were purchased from Sigma and Gibco and were further purified by gel filtration and ion exchange chromatography. The assay was performed as described previously [9]. ^125^I-HGF (NEN) in binding buffer (PBS containing 2% BSA) were added to the microtitre plates and incubated for 30 min at room temperature (RT) before washing and counting to determine bound radioactivity. SPR analysis was performed on the BIAcore X (Biacore Herts UK) as described previously [9]. HGF (30–500 nM) was injected across the FN 70 kDa fragment immobilised on a CM5 chip in HEPES saline (pH 7.4) supplemented with 1 mM MgCl_2_, 2 mM CaCl_2 _and the sensograms recorded. The data was analysed by the ASSAY programme (Biosoft, UK) in order to determine the EC_50 _value and K_d_.

### Migration and proliferation assays

Human dermal microvessel endothelial cells (HMVEC) were maintained in EBM-2 growth medium (Clonetics Corp). Migration studies were carried out essentially as described previously [9] using serum starved Calcein AM-loaded HMVEC in a modified Boyden chamber assay using Fluorblok transwell chambers (BD Bioscience) as described by the manufacturer. Cell migration was detected by fluorescence measurement (within the lower chamber compartment). Membranes of transwell chambers were coated with either FN or VN or collagen-1 (10 μg/ml) overnight at 4°C and preliminary experiments were performed to assess the optimal dosage of both HGF and ECM protein. With antibody inhibition studies, the transwell chamber was coated with poly-L-lysine (Sigma) to facilitate cell attachment to the filters as opposed to adhesion using ECM glycoproteins. HMVEC were pre-treated with α_v_β_3 _and α_5_β_1 _integrin blocking antibodies for 30 min at room temperature prior to application to the upper transwell chamber. The levels of cell adhesion to ECM-coated transwell filters were determined by allowing HGF-stimulated HMVEC to adhere to transwells (coated overnight with ECM glycoprotein (10 μg/ml) and then blocked by incubation in 3.5 mg/ml BSA in basal culture medium) for 1 hour at room temperature followed by extensive washes with basal culture medium. The remaining cells were measured using a fluorescence plate reader (measuring fluorescence in the upper transwell compartment). For proliferation experiments, cell division was measure by fluorescence labeling of DNA (CyQuant, Molecular Probes). HMVEC was plated on poly-D-lysine coated 48-well plates and cultured overnight in MCDB-131 medium containing 5% FBS. After washing plates with PBS, endothelial cells were then cultured in MCDB-131 medium + 0.1% FBS containing HGF (10 ng/ml) in the presence or absence of VN, FN or Collagen-1 (10 μg/ml). Cells incubated for 48 h and HGF/ECM was added every 24 hours. Cell proliferation was quantified using a fluorescence plate reader.

### Phosphorylation analysis and ras activation

HMVEC were assessed for the activation profiles of Erk1/2 and Akt using phosphospecific antibodies (Cell Signalling Technology) to Erk (Thr^202^/Tyr^204^) and Akt (Ser^473^) respectively by Western blotting. These studies were performed with both cells in suspension and with adherent populations. Cells were grown to 80% confluence and serum starved for 2 hours prior to harvesting. Cells were resuspended in serum-free MCDB-131 medium (BioWhittaker) supplemented with 0.1% BSA (resuspension buffer) at a concentration of 1–5 × 10^6 ^cells /ml. The cell suspensions were challenged 10 ng/ml HGF supplemented with 2 μg/ml collagen-1, or FN or VN for various durations ranging from 2 to 120 min at room temperature. Cells were harvested by centrifugation at 4°C and lysed in 10 mM Tris pH 7.4, 145 mM NaCl supplemented with 0.1% Triton X-100 and protease inhibitors. For inhibitor studies, serum-starved HMVEC suspensions were pre-treated with the inhibitor for 45 min prior to stimulation with HGF and ECM molecules for a further 60 min at room temperature. The cells were pelleted, washed in ice-cold resuspension buffer without BSA and lysed in a lysis buffer containing 1% (v/v) Triton X-100. Cell lysates were analysed by Western blotting using protocols specific for the phosphospecific antibodies according to the manufacturer's recommendations. Blots were cut along appropriate marker divides and probed with antibodies to phopho Erk 1/2 and Akt (Ser^473^) (Cell Signaling Technologies) simultaneously. For GTP-Ras pull down assays, serum-starved HMVEC were stimulated with HGF and ECM molecules for a desired time point and the cells were spun down and washed in ice-cold resuspension buffer without BSA. Cell pellets were lysed in MLB buffer (25 mM HEPES pH 7.5, 150 mM NaCl, 1 % Igepal CA-630, 10% Glycerol, 25 mM NaF, 10 mM MgCl_2_, 1 mM EDTA, 1 mM sodium orthovanadate and protease inhibitor cocktail) and 500 μg of cell lysate was mixed with a 10 μl suspension of RBD-Sepharose (Upstate Biotechnology) for each reaction at 4°C for 60 min. Sepharose beads were spun down and washed in MLB prior to solubilization and analysis by Western blotting probing for Ras using a monoclonal antibody (Upstate Biotechnology). For Ras inhibition studies cells were pre-incubated with FPT-III (100 μM) and GGTI (2 μM) (approx 40 × IC_50 _values) for 45 min at room temperature prior to cell stimulation for 60 min with HGF and ECM.

### Met-Integrin immunoprecipitation

Human microvessel endothelial cells (HMVEC) in serum-free MCDB-131 medium (BioWhittaker) supplemented with 0.1% BSA were plated on collagen, FN and VN coated petri dishes in the absence or presence of HGF (50 ng/ml) for 15 min to 1 hour at room temperature. Cells were then harvested as described previously [9] and integrin immunoprecipitation was performed with monoclonal antibodies (Chemicon) to α_2_β_1 _(clone JBS2), α_5_β_1 _(clone JBS5) and α_v_β_3 _clone (LM609). After analysis by SDS-PAGE and protein transfer, the blot was then probed with a monoclonal to Met (clone DL-21, Upstate Biotechnology) and developed by chemiluminescence. For Met tyrosine phosphorylation analysis, cells were stimulated with HGF alone or HGF-FN and HGF-VN complexes for various time points ranging from 15 mins to 2 hours at room temperature. Lysed samples were immunoprecipitated with a polyclonal anti-phosphoMet antibody (Cell Signalling) and the immune complexes analysed by SDS-PAGE and Western blotting using a monoclonal ant-Met antibody (Upstate). Met was visualised using chemiluminescence technology (Pierce).

### Immunoprecipitation of FN-HGF and VN-HGF complex from platelet supernatants

Supernatants from non-stimulated and thrombin-stimulated washed platelet suspensions were prepared as previously described [9]. Supernatants were immunoprecipated with an antibody to FN or VN (Chemicon) or an isotype matched control reagent (IgG). Following SDS-PAGE and immunoblotting, HGF was detected with a polyclonal antibody (Santa Cruz) by chemiluminescent development.

## Abbreviations

HGF, hepatocyte growth factor, FN fibronectin, VN vitronectin, Col-1, collagen-1, HMVEC, human microvessel endothelial cells.

## Authors' contributions

SR, study design, HMVEC migration studies, SPR ligand binding analysis, HMVEC signalling studies, co-immunoprecipitation studies (integrin-Ras), manuscript preparation. ESW, study design, HMVEC proliferation studies, solid-phase ligand binding studies, co-imunoprecipitation (integrin-Met), manuscript preparation. YMP, study design, co-imunoprecipitation studies (HGF-FN/VN). JM, protein purification and preparation, KVP, GTP-Ras pull-down assays, RS, technical support. MS, study design.

## References

[B1] Ausprunk DH, Folkman J (1977). Migration and proliferation of endothelial cells in preformed and newly formed blood vessels during tumor angiogenesis. Microvasc Res.

[B2] Carmeliet P, Jain RK (2000). Angiogenesis in cancer and other diseases. Nature.

[B3] Rafii S, Lyden D, Benezra R, Hattori K, Heissig B (2002). Vascular and haematopoietic stem cells: novel targets for anti-angiogenesis therapy?. Nat Rev Cancer.

[B4] Stupack DG, Cheresh DA (2003). Apoptotic cues from the extracellular matrix: regulators of angiogenesis. Oncogene.

[B5] Schneller M, Vuori K, Ruoslahti E (1997). Alphavbeta3 integrin associates with activated insulin and PDGFbeta receptors and potentiates the biological activity of PDGF. Embo J.

[B6] Soldi R, Mitola S, Strasly M, Defilippi P, Tarone G, Bussolino F (1999). Role of alphavbeta3 integrin in the activation of vascular endothelial growth factor receptor-2. Embo J.

[B7] Woodard AS, Garcia-Cardena G, Leong M, Madri JA, Sessa WC, Languino LR (1998). The synergistic activity of alphavbeta3 integrin and PDGF receptor increases cell migration. J Cell Sci.

[B8] Miyamoto S, Teramoto H, Gutkind JS, Yamada KM (1996). Integrins can collaborate with growth factors for phosphorylation of receptor tyrosine kinases and MAP kinase activation: roles of integrin aggregation and occupancy of receptors. J Cell Biol.

[B9] Wijelath ES, Murray J, Rahman S, Patel Y, Ishida A, Strand K, Aziz S, Cardona C, Hammond WP, Savidge GF, Rafii S, Sobel M (2002). Novel vascular endothelial growth factor binding domains of fibronectin enhance vascular endothelial growth factor biological activity. Circ Res.

[B10] Wijelath ES, Rahman S, Murray J, Patel Y, Savidge G, Sobel M (2004). Fibronectin promotes VEGF-induced CD34 cell differentiation into endothelial cells. J Vasc Surg.

[B11] Xin X, Yang S, Ingle G, Zlot C, Rangell L, Kowalski J, Schwall R, Ferrara N, Gerritsen ME (2001). Hepatocyte growth factor enhances vascular endothelial growth factor-induced angiogenesis in vitro and in vivo. Am J Pathol.

[B12] Van Belle E, Witzenbichler B, Chen D, Silver M, Chang L, Schwall R, Isner JM (1998). Potentiated angiogenic effect of scatter factor/hepatocyte growth factor via induction of vascular endothelial growth factor: the case for paracrine amplification of angiogenesis. Circulation.

[B13] Heeschen C, Dimmeler S, Hamm CW, Boersma E, Zeiher AM, Simoons ML (2003). Prognostic significance of angiogenic growth factor serum levels in patients with acute coronary syndromes. Circulation.

[B14] Blystone SD, Slater SE, Williams MP, Crow MT, Brown EJ (1999). A molecular mechanism of integrin crosstalk: alphavbeta3 suppression of calcium/calmodulin-dependent protein kinase II regulates alpha5beta1 function. J Cell Biol.

[B15] Kim S, Harris M, Varner JA (2000). Regulation of integrin alpha vbeta 3-mediated endothelial cell migration and angiogenesis by integrin alpha5beta1 and protein kinase A. J Biol Chem.

[B16] Gerber HP, McMurtrey A, Kowalski J, Yan M, Keyt BA, Dixit V, Ferrara N (1998). Vascular endothelial growth factor regulates endothelial cell survival through the phosphatidylinositol 3'-kinase/Akt signal transduction pathway. Requirement for Flk-1/KDR activation. J Biol Chem.

[B17] Rodriguez-Viciana P, Warne PH, Dhand R, Vanhaesebroeck B, Gout I, Fry MJ, Waterfield MD, Downward J (1994). Phosphatidylinositol-3-OH kinase as a direct target of Ras. Nature.

[B18] Rubio I, Rodriguez-Viciana P, Downward J, Wetzker R (1997). Interaction of Ras with phosphoinositide 3-kinase gamma. Biochem J.

[B19] Rodriguez-Viciana P, Warne PH, Vanhaesebroeck B, Waterfield MD, Downward J (1996). Activation of phosphoinositide 3-kinase by interaction with Ras and by point mutation. Embo J.

[B20] Rodriguez-Viciana P, Warne PH, Khwaja A, Marte BM, Pappin D, Das P, Waterfield MD, Ridley A, Downward J (1997). Role of phosphoinositide 3-OH kinase in cell transformation and control of the actin cytoskeleton by Ras. Cell.

[B21] Weimar IS, Miranda N, Muller EJ, Hekman A, Kerst JM, de Gast GC, Gerritsen WR (1998). Hepatocyte growth factor/scatter factor (HGF/SF) is produced by human bone marrow stromal cells and promotes proliferation, adhesion and survival of human hematopoietic progenitor cells (CD34+). Exp Hematol.

[B22] Beviglia L, Kramer RH (1999). HGF induces FAK activation and integrin-mediated adhesion in MTLn3 breast carcinoma cells. Int J Cancer.

[B23] Hood JD, Frausto R, Kiosses WB, Schwartz MA, Cheresh DA (2003). Differential alphav integrin-mediated Ras-ERK signaling during two pathways of angiogenesis. J Cell Biol.

[B24] Morales-Ruiz M, Lee MJ, Zollner S, Gratton JP, Scotland R, Shiojima I, Walsh K, Hla T, Sessa WC (2001). Sphingosine 1-phosphate activates Akt, nitric oxide production, and chemotaxis through a Gi protein/phosphoinositide 3-kinase pathway in endothelial cells. J Biol Chem.

[B25] Purdie KJ, Whitley GS, Johnstone AP, Cartwright JE (2002). Hepatocyte growth factor-induced endothelial cell motility is mediated by the upregulation of inducible nitric oxide synthase expression. Cardiovasc Res.

[B26] Kawasaki K, Smith RSJ, Hsieh CM, Sun J, Chao J, Liao JK (2003). Activation of the phosphatidylinositol 3-kinase/protein kinase Akt pathway mediates nitric oxide-induced endothelial cell migration and angiogenesis. Mol Cell Biol.

[B27] Deregibus MC, Buttiglieri S, Russo S, Bussolati B, Camussi G (2003). CD40-dependent activation of phosphatidylinositol 3-kinase/Akt pathway mediates endothelial cell survival and in vitro angiogenesis. J Biol Chem.

[B28] Steinle JJ, Meininger CJ, Forough R, Wu G, Wu MH, Granger HJ (2002). Eph B4 receptor signaling mediates endothelial cell migration and proliferation via the phosphatidylinositol 3-kinase pathway. J Biol Chem.

[B29] Ma H, Calderon TM, Kessel T, Ashton AW, Berman JW (2003). Mechanisms of hepatocyte growth factor-mediated vascular smooth muscle cell migration. Circ Res.

[B30] Holgado-Madruga M, Moscatello DK, Emlet DR, Dieterich R, Wong AJ (1997). Grb2-associated binder-1 mediates phosphatidylinositol 3-kinase activation and the promotion of cell survival by nerve growth factor. Proc Natl Acad Sci U S A.

[B31] Trusolino L, Bertotti A, Comoglio PM (2001). A signaling adapter function for alpha6beta4 integrin in the control of HGF-dependent invasive growth. Cell.

[B32] Zhang YW, Su Y, Volpert OV, Vande Woude GF (2003). Hepatocyte growth factor/scatter factor mediates angiogenesis through positive VEGF and negative thrombospondin 1 regulation. Proc Natl Acad Sci U S A.

